# Prevalence of Pure Red Cell Aplasia Following Major ABO-Incompatible Hematopoietic Stem Cell Transplantation

**DOI:** 10.3389/fimmu.2022.829670

**Published:** 2022-02-11

**Authors:** Panpan Zhu, Yibo Wu, Dawei Cui, Jimin Shi, Jian Yu, Yanmin Zhao, Xiaoyu Lai, Lizhen Liu, Jue Xie, He Huang, Yi Luo

**Affiliations:** ^1^Bone Marrow Transplantation Center, The First Affiliated Hospital, Zhejiang University School of Medicine, Hangzhou, China; ^2^Liangzhu Laboratory, Zhejiang University Medical Center, Hangzhou, China; ^3^Institute of Hematology, Zhejiang University, Hangzhou, China; ^4^Zhejiang Province Engineering Laboratory for Stem Cell and Immunity Therapy, Hangzhou, China; ^5^Department of Blood Transfusion, The First Affiliated Hospital, Zhejiang University School of Medicine, Hangzhou, China

**Keywords:** pure red cell aplasia, major ABO-incompatible transplantation, haploidentical donor, allogeneic hematologic stem cell transplantation, isohemagglutinin

## Abstract

**Background:**

Pure red cell aplasia (PRCA) is one of the important complications in major ABO-incompatible allogeneic hematopoietic stem cell transplantation (HSCT). The established pathogenic factor of PRCA is the persistence of high anti-donor isohemagglutinins. As previously verified, the conditioning regimen and donor type were the factors associated with the development of PRCA in the small-sized studies. Currently, the prevalence, risk factors, and prognosis of PRCA are still worth studying to provide evidence.

**Methods:**

We conducted a prospective nested case-control study to determine the prevalence, donor-related factors, and the outcomes of PRCA following major ABO-incompatible transplantation. A total of 469 patients who underwent ABO-incompatible grafts were observed.

**Results:**

None of the patients were diagnosed with PRCA with minor or bidirectional ABO-incompatible HSCT. Thirteen of the187 patients (7%; 95% confidence interval [CI], 3.9%–11.9%) developed PRCA following major ABO-incompatible HSCT. Eleven of the 13 patients with PRCA recovered entirely. Donor type was an independent factor associated with post-HSCT PRCA (odds ratio [OR]=0.030; 95% CI, 0.003–0.321; *P*=0.004). The cumulative incidence rates of post-HSCT PRCA in the context of major ABO-incompatible HSCT were 0.8%, 13.1%, and 27.2% for the haploidentical donor (HID), unrelated donor, and matched related donor, respectively. No significant influence of PRCA on transplantation outcomes was observed.

In conclusion, post-HSCT PRCA is a rare and less threatening complication in major ABO-incompatible HSCT. The majority of patients with PRCA could recover. Additionally, HIDs for recipients may have a low risk of post-HSCT PRCA. This trial was registered at www.chictr.org.cn (#ChiCTR2000041412).

## Introduction

ABO-blood group incompatibility occurs in 25% to 50% of human leukocyte antigen-matched hematopoietic stem cell transplantation (HSCT) (1). Although ABO incompatibility has no effect on transplantation survival and relapse of underlying disease (2–4), hemolysis, the delayed red blood cell engraftment, and pure red cell aplasia (PRCA) have potential clinical consequences following ABO-incompatible transplantation (5, 6).

PRCA is a critical complication in patients undergoing major ABO-incompatible HSCT, which may result in significant iron overload and increase non-relapse mortality after HSCT (7). The incidence rate of PRCA after ABO-mismatched transplantation ranged from 7% to 30% (7–9). The mechanism of post-HSCT PRCA was not clear enough until now. It was reported that the persistence of anti-donor isohemagglutinins (ISO) produced by recipient plasma cells may contribute to post-HSCT PRCA (6, 10). However, a few patients with a low pre-HSCT isohemagglutinin titer are likely to develop post-HSCT PRCA (11), which may be attributed to the rebound of anti-donor ISO and the post-HSCT transfusion of recipient-type RBC (12–14).

To the best of our knowledge, patients with type O blood who received the grafts from the donors with type A blood were at high risk for post-HSCT PRCA (8, 15, 16). Compared to type B blood antigen, there is more intensive type A blood group antigen on red blood cell (RBC) membrane, which leads to an increased complement-fixing capacity of red-cell-bound anti-A under the circumstance of a high level of anti-donor isohemagglutinins (17–19). Besides, delayed donor erythropoiesis was more common in reduced-intensity HSCT, which was associated with prolonged persistence of host anti-donor isohemagglutinins (6). In an ABO-incompatible HSCT study using 296 matched related donors (MSDs) and 420 matched unrelated donors (URDs), it was demonstrated that the graft-versus-plasma cell effect plays an essential role in the disappearance rate of anti-donor isohemagglutinins (20). However, this large study lacked of the information on post-HSCT PRCA. A previous study on post-HSCT PRCA was restricted to small sample size (21). Hence, the prevalence, risk factors, and prognosis of PRCA are still worth studying to provide evidence.

Here, a prospective nested case-control study was conducted to determine the prevalence, donor-related factors, and the outcomes of PRCA following major ABO-incompatible transplantation.

## Methods

### Patient

**Figure 1:**
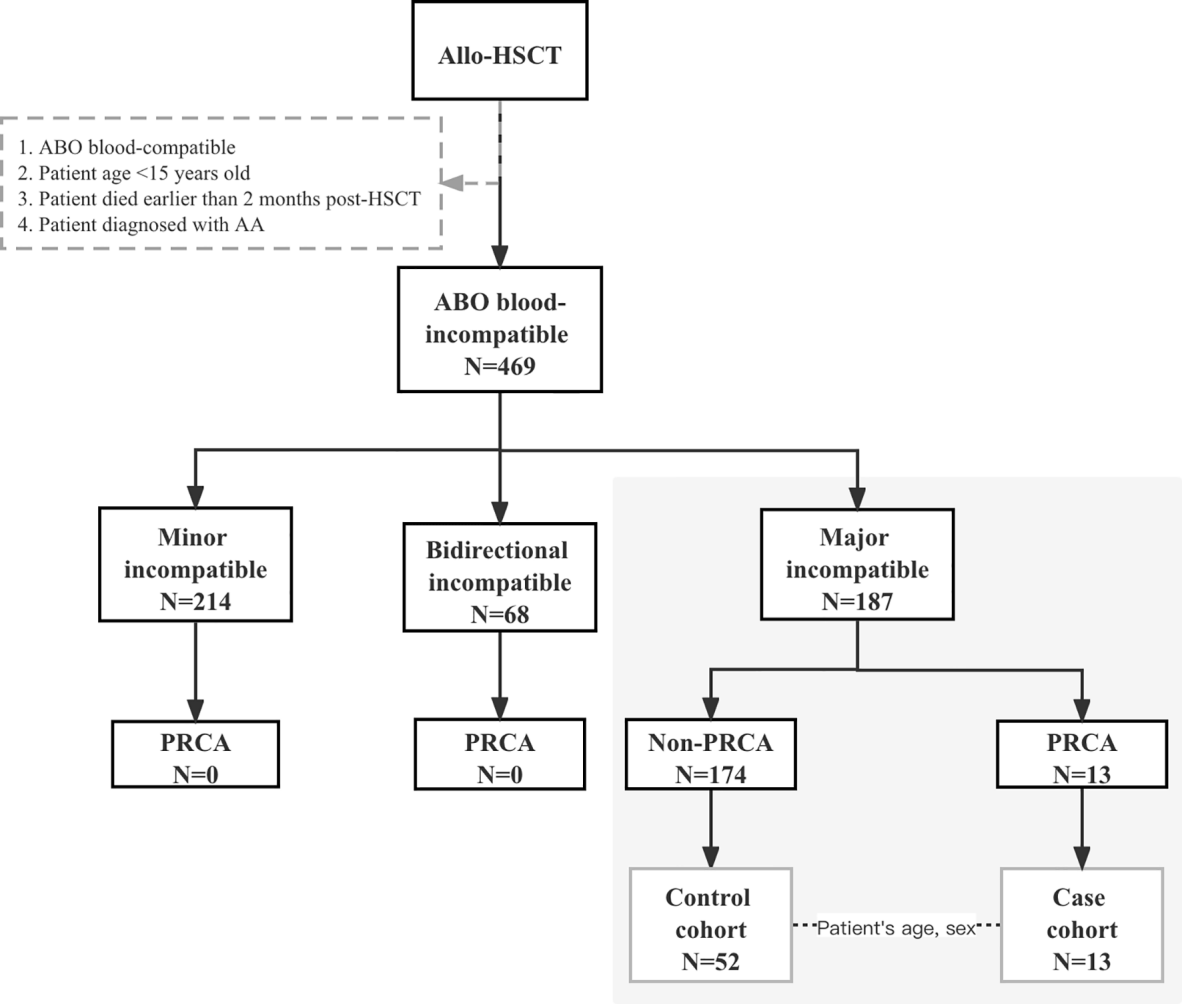
Diagram of patients with ABO-incompatible hematopoietic stem cell transplantation.

Patients participated in this prospective observational study at the First Affiliated Hospital of Zhejiang University School of Medicine between August 1, 2014, and June 30, 2020. The final day of the last follow-up for all the surviving patients was October 31, 2020. Patients who underwent ABO-incompatible HSCT consecutively during this period were observed, and the study cohort comprised patients who received major ABO-incompatible grafts, as shown in **Figure 1**. Patients aged 15 years below, diagnosed with aplastic anemia before HSCT, and those who died within 2 months post-HSCT were excluded. For each PRCA case, four matched controls without PRCA were randomly selected from the same cohort at the onset of PRCA and were matched according to the patient’s age (± 5years) and patient’s sex. All patients provided their written informed consent in accordance with the Declaration of Helsinki. This study was approved by the Ethics Review Committee of the First Affiliated Hospital of Zhejiang University School of Medicine.

### Transplantation Procedure

Patients received myeloablative conditioning (MAC) or reduced-intensity conditioning (RIC). MAC consisted of busulfan and cyclophosphamide (BUCY) or modified BUCY according to the disease features and transplantation patterns described previously (22). RIC consisted of fludarabine and busulfan or fludarabine and cyclophosphamide. Graft-versus-host disease (GVHD) prophylaxis consisting of cyclosporin A, methotrexate, and low-dose mycophenolate mofetil was administered to patients. Rabbit anti-thymocyte globulin (ATG; Thymoglobulin, Genzyme, Cambridge, MA, USA) or anti-T-lymphocyte globulin (ATG-F; Fresenius, Bad Homburg, Germany) was administered. Grafts derived from peripheral blood were mobilized with recombinant human granulocyte colony-stimulating factor (5–7.5 μg/kg/d; Filgrastim; Kirin, Japan) for 5 to 6 consecutive days from day –4. All patients received unmanipulated grafts.

### ABO Blood Group and Anti-Donor Isohemagglutinins

The whole blood collected in ethylenediaminetetraacetic acid was used to determine the ABO forward/reverse typing. Anti-donor isohemagglutinins (ISO, anti-A IgG or IgM, anti-B IgG or IgM) were determined by incubating a 3% standard A and B erythrocyte suspension (RBC kits for human ABO reverse typing, Kinghaw, China) in saline with twofold serial dilutions of serum followed by centrifugation. Anti-donor isohemagglutinin titers were scored with a microscope for IgM and with a macroscope for IgG using an anti-human globulin test card (D.G. Gel Coombs, Diagnostic Grifols, S.A., Spain). Anti-donor isohemagglutinin titers were monitored at the time of stem cell transfusion (pre-HSCT ISO) and at 4 months post-transplantation (post-HSCT ISO) in 28 patients among this cohort, which comprised 7 patients with PRCA and 21 patients without PRCA.

### Definitions and Endpoints

Post-transplantation PRCA diagnosis was established if persistently severe normocytic anemia, reticulocytopenia, and absence of erythroblasts from otherwise normal bone marrow occurred for more than 60 days post-HSCT, which occurred in the absence of leukemia relapse, drug toxicity, or infection (11, 23). Primary post-HSCT PRCA was distinguished before the initial engraftment of red cells; otherwise, it was considered a secondary post-HSCT PRCA. The titer index was defined as the dilution time of the anti-donor isohemagglutinins. Neutrophil engraftment was defined as the first day of 3 consecutive days of an absolute neutrophil count > 0.5 × 10^9^/L. Platelet engraftment was defined as the first 7 consecutive days with a platelet count > 20 × 10^9^/L without transfusion support. Disease classification before transplantation was based on the established Refined Disease Risk Index (DRI-R) (24). The primary endpoint of this study was the cumulative incidence of PRCA. Engraftment, red cell transfusion, acute GVHD and chronic GVHD, relapse rate, non-relapse mortality (NRM, death from any cause exclusive of leukemia), overall survival (OS), and disease-free survival (DFS) were also observed.

### Statistical Methods

Statistical analysis was performed using the Statistical Package for the Scoial Sciences (SPSS) version 26.0 software (SPSS, Chicago, IL, USA) and R language statistical software (http://www.r-project.org). Continuous variables are summarized as medians and ranges. Differences between cohorts normally distributed with homogeneity of variance were analyzed using Student’s t-test. Otherwise, differences were tested using the Wilcoxon rank-sums test. Pearson’s chi-squared test (T≥5 and n≥40), chi-squared test with continuity correction (1≤T<5 and n≥40), and Fisher’s exact test were used to compare categorical variables. The cumulative incidence of PRCA was estimated using the cumulative incidence function, with death as a competing event. Conditional logistic regression was used to examine the correlation between risk factors and PRCA occurrence. A forward likelihood ratio method was used. The Kaplan–Meier method was used to estimate the 2-year OS and disease-free survival by comparing patients with and without PRCA. Gray’s competing risk method was used to compute acute GVHD, chronic GVHD, and the 2-year NRM cumulative incidence curve. Cox regression models were used to estimate the hazard ratio for transplantation survival. All tests were two-tailed, and *P* values <0.05 were considered statistically significant.

## Results

### Cohort Characteristic

A total of 469 patients underwent ABO-mismatched allogeneic HSCT, of which 187 patients received major ABO-incompatible grafts. In the major ABO-mismatched group, the median age was 35.5 (range, 15–67) years and 37 (range, 13–56) years for patients and donors, respectively. Patients with PRCA were only observed in the major ABO-mismatched group, where 13 patients (7%; 95% confidence interval [CI], 3.9%–11.9%) were diagnosed with post-HSCT PRCA. The cohort characteristics and risk factors of PRCA in major ABO-incompatible transplantations are shown in **Table 1**. Fifty-two major ABO-incompatible transplantation recipients were selected as controls. No significant difference was observed between the two groups in terms of patient’s age, patient’s sex, disease, donor’s sex, conditioning regimens, and the type of anti-donor isohemagglutinins. However, the DRI-R, donor type, donor’s age, and GVHD prophylaxis were significantly different between the two groups.

**Table 1:** Characteristics of the nest case-control cohort.

Characteristic	PRCA (N = 13)^*^	Non-PRCA (N = 52)^*^	P-value
Patient sex, female/male, n	9/4	36/16	1.000
Patient age, median (range), years	46 (27-55)	45 (25-58)	0.954
Disease			0.642
AML/MDS	7	34	
ALL	5	13	
Other	1	5	
**DRI-R**			**0.018^#^**
Low/Int	11	25	
High/very high	2	27	
Donor sex, female/male, n	6/7	17/35	0.559
**Donor age, median (range), years**	40 (14-53)	26 (13-55)	**0.022^#^**
**Donor type**			**<0.001^##^**
MSD	9	7	
HID	1	40	
URD	3	5	
Intensity of conditioning regimen			1.000
MAC	12	48	
RIC	1	4	
**ATG for GVHD prophylaxis**			**<0.001^##^**
Yes	6	48	
No	7	4	
Donor-recipient blood type			0.524
A–O	6	19	
Other	7	33	
CD34+ cells, median (range), ×10^6^/kg	5.72 (3.05-13.50)	5.69 (2.03-15.09)	0.973
MNC, median (range), ×10^8^/kg	13.48 (7.56-43.90)	15.20 (5.65-45.76)	0.820
Red cell transfusion at d100, median (range), U	6 (0-35)	4.5 (0-33)	0.451
Acute GVHD at d100, n			
Grades II-IV	2	14	0.614
Grades III-IV	0	5	0.561
Moderate-severe chronic GVHD, n	1	8	0.788
Median follow-up (range), Mo	24.6 (2.4-74.1)	27.7 (3.5-65.4)	0.825

PRCA, pure red cell aplasia; AML, acute myelocytic leukemia; ALL, acute lymphoblastic leukemia; MDS, myelodysplastic syndrome; DRI-R, refined disease risk index; HID, haploidentical donor; MSD, matched sibling donor; URD, unrelated donor; GVHD, graft versus host disease; MAC, myeloablative conditioning; RIC, reduced intensity conditioning.

*For each PRCA case, 4 controls were selected at random from the same cohort.

^#^the P-value < 0.05; ^##^the P-value < 0.01.

### Occurrence of *Post*-HSCT PRCA

**Table 2** illustrated the detailed information of patients with post-HSCT PRCA. The post-HSCT PRCA group comprised seven patients with primary post-HSCT PRCA and six patients with secondary post-HSCT PRCA. A significant difference of underlying disease distribution was observed between the two groups, and there was no difference on patient age, patient sex, donor age, donor sex, donor type, and ATG usage between the primary and secondary PRCA groups (**Supplemental Table 1**). All patients with primary PRCA were initially identified the first month post-HSCT. The median occurrence of secondary PRCA was 60 (range, 45–114) days post-HSCT. In the primary post-HSCT PRCA subgroup, one patient had persistent pancytopenia, and died from transplantation-associated thrombotic microangiopathy and lung infection 73 days post-HSCT. The median recovery time of primary PRCA was 125.5 (range, 116–300) days post-HSCT in the remaining six patients with PRCA. In the subgroup of secondary post-HSCT PRCA, the median PRCA diagnosis time was 60 (range, 45–114) days post-HSCT. One patient with secondary PRCA was diagnosed 58 days post-HSCT and experienced leukemia relapse 90 days post-HSCT. The median interval from diagnosis to recovery of secondary PRCA was 158 (range, 66–777) days in the remaining five patients with PRCA. Up to the last follow-up, 11of the 13 patients with PRCA recovered from PRCA entirely and achieved HSCT success. Two patients with PRCA received blood transfusion support only, and three patients were treated with intravenous immunoglobulin (IVIG) and blood transfusion. Therapeutic plasma exchange (TPE) was provided for another six patients, of which two patients were provided additional interventions, such as eltrombopag, rituximab, and donor lymphocyte infusion. The immunosuppressive agent was tapered slowly once the patient was diagnosed with PRCA.

**Table 2:** Characteristics of patients with pure red cell aplasia.

UPN	Disease /DRI-r	Donor type/graft	Age P/D	Sex P/D	ABO blood type P/D	Conditioning regimen/GVHD prophylaxis	aGVHD at d100	initial time (days)	PRCA Course (days)	Iso-titer (diagnosis)	Iso-titer (resolution)	Treatment of PRCA	Outcome
**1**	AML/Int	MSD/PB	46/51	F/F	O/A	MAC	Grade 2	primary	126	IgG 1:128	IgG 1:1	IVIG/transfusion	Alive, CR
						CsA/MMF/MTX				IgM 0	NA		
**2**	AML/Int	MSD/PB	30/39	M/F	O/B	MAC	Absent	62	66	IgG 0	IgG 0	IVIG/transfusion	Alive, CR
						CsA/MMF/MTX				IgM 1:1	IgM 0		
**3**	AML/Int	MSD/PB	50/53	F/M	O/B	MAC	Absent	45	777	IgG 1:512	IgG 1:1	IVIG/transfusion	Alive, CR
						CsA/MMF/MTX				IgM 1:8	NA		
**4**	AML/Int	MSD/PB	53/50	M/M	O/B	MAC	Absent	58	NA	IgG 1:512	Relapse*	transfusion	AML relapse, demise
										IgM 1:128			
						CsA/MMF/MTX							
**5**	ALL/Int	MSD/PB	52/45	F/M	O/B	MAC	Absent	primary	300	IgG 1:1024	IgG 1:64	TPE/IVIG/transfusion	Alive, CR
						CsA/MMF/MTX				IgM 1:16	IgM 1:8		
**6**	HLH	MSD/PB	37/27	F/F	O/A	RIC	Absent	97	664	IgG 1:64	IgG 1:64	TPE/RTX/Eltrombopag/transfusion	Alive, CR
						CsA/MTX+ATG				IgM 1:2	IgM 1:2		
**7**	ALL/Int	HID/PB	51/14	M/F	O/A	MAC	Absent	primary	116	IgG 1:128	IgG 1:64	Transfusion	Alive, CR
						CsA/MMF/MTX +ATG				IgM 1:16	IgM 1:4		
**8**	MDS/Int	URD/PB	49/36	F/F	O/B	MAC	Grade 2	primary	NA	IgG 1:1024	NRM*	Transfusion	Viremia, demise
						CsA/MMF/MTX +ATG							
										IgM 1:2			
**9**	ALL/Int	MSD/PB	28/26	F/M	O/A	MAC	Absent	primary	195	IgG 1:128	IgG <1:64	TPE/DLI/Eltrombopag/transfusion	Alive, CR
										IgM 1:32	IgM 1:4		
						CsA/MTX							
**10**	ALL/Int	MSD/PB	41/50	F/M	O/B	MAC	Absent	primary	123	IgG 1:64	IgG 1:64	TPE/transfusion	Alive, CR
						CsA/MMF/MTX				IgM 1:16	IgM 1:1		
**11**	AML/High	URD/PB	40/43	F/M	O/B	MAC	Absent	114	158	IgG 1:64	IgG 1:64	Transfusion	Alive, CR
										IgM 1:2	IgM 1:1		
						CsA/MMF/MTX +ATG							
**12**	AML/High	URD/PB	55/32	M/M	O/A	MAC	Absent	55	82	IgG 1:64	IgG 1:8	TPE/transfusion	Alive, CR
										IgM 1:4	IgM 1:1		
						CsA/MMF/MTX +ATG							
**13**	ALL/Int	MSD/PB	38/40	F/F	O/A	MAC	Absent	primary	125	IgG 1:64	IgG 1:64	TPE/IVIG/transfusion	Alive, CR
						CsA/MTX +ATG				IgM 1:4	IgM 1:1		

*the data was unavailable because of leukemia relapse and non-relapse mortality (NRM).

AML, acute myelocytic leukemia; ALL, acute lymphoblastic leukemia; MDS, myelodysplastic syndrome; HLH, hemophagocytic lymphohistiocytosis; P, patient; D, donor; F, female; M, male; TPE, therapeutic plasma exchange; IVIG, intravenous immunoglobulin; RTX, rituximab; DRI-R, refined disease risk index; HID, haploidentical donor; MSD, matched sibling donor; URD, unrelated donor.

### Pure Red Cell Aplasia (PRCA) Prevalence in the Entire Group

**Figure 2:**
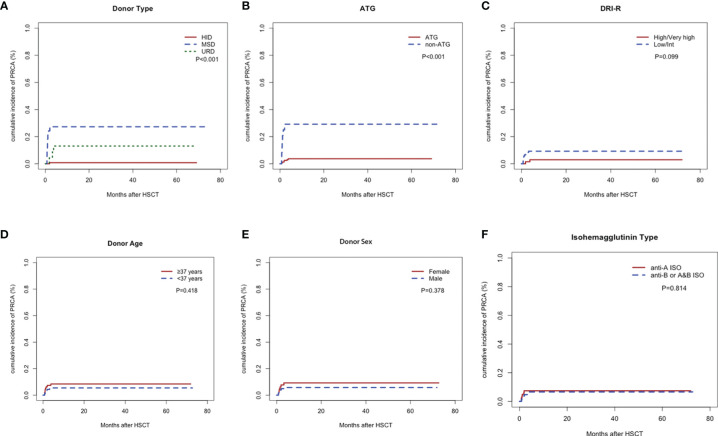
Cumulative incidence rates of pure red cell aplasia. Donor type **(A)**, anti-thymocyte globulin **(B)**, Refined Disease Risk Index **(C)**, donor age **(D)**, donor sex **(E)**, and anti-donor isohemagglutinins type **(F)**.

**Figure 2** illustrates the cumulative incidence rates of PRCA in the different subgroups of 187 patients with major ABO-incompatible HSCT. The cumulative incidence rates of post-HSCT PRCA were 0.8%, 13.1%, and 27.2% for HID, URD, and MSD, respectively (*P*<0.001). A significant difference in PRCA rate was found between patients who received ATG and those who did not receive ATG (3.7% versus 29.2%, *P*<0.001). Patients with high/very high disease status tended to have lower PRCA rates than those with low/Int disease status, but the difference (*P*=0.099) did not reach statistical significance. No difference in PRCA rate was observed between groups with respect to donor age, donor sex, and the anti-donor isohemagglutinins type.

### Risk Factors of PRCA

According to the cohort study of 65 patients receiving major ABO-incompatible peripheral blood grafts, the factors associated with post-HSCT PRCA were DRI-R, donor age, donor type, and ATG in the univariate analysis (**Table 3**). However, in the multivariate analysis using conditional logistic regression test, donor type was the only independent factor associated with post-HSCT PRCA (odds ratio=0.030; 95% CI, 0.003–0.321; *P*=0.004). Patients using MSDs or URDs had a significantly higher post-HSCT PRCA rate than those using HIDs (2.4% versus 50.0%, *P*<0.001; **Supplemental Figure 1A**). Patients with high/very high disease status tended to have a lower risk of post-HSCT PRCA occurrence than those with low/Int disease status (6.9% versus 30.6%, *P*=0.016; **Supplemental Figure 1B**), although the difference was not statistically significant (*P*=0.065).

**Table 3:** Univariate and multivariate analyses of risk factors for pure red cell aplasia in the nested case control cohort.

	Univariate analysis			Multivariate analysis		
	OR	95% CI	*P*-value	OR	95% CI	*P*-value
DRI-R (High/very high vs Low/Int)	0.167	0.033-0.829	0.029	0.100	0.009-1.151	0.065
Donor age (≥37 vs <37)	4.959	1.221-20.134	0.025	NA	NA	0.610
Donor type (HID vs MSD/URD)	0.037	0.005-0.295	0.002	0.030	0.003-0.321	0.004
ATG (Yes vs No)	0.085	0.017-0.415	0.002	NA	NA	0.464
Donor-recipient blood type (A–O vs other)	NA	NA	0.525	–	–	–
Donor gender (Male vs female)	NA	NA	0.332	–	–	–

DRI-R, refined disease risk index; HID, haploidentical donor; MSD, matched sibling donor; URD, unrelated donor; NA, not available.

Meanwhile, we performed the univariate and multivariate analysis for post-PRCA using Cox proportional-hazards model in the entire cohort of 187 patients with major ABO-mismatched HSCT. The baseline characteristics of the entire cohort was shown in **Supplemental Table 2**. As shown in **Table 4**, the patient age (*P*=0.022), ATG (*P*<0.001), and donor type (*P*<0.001) had an impact on the development of PRCA in the univariate analysis. Donor type was the only independent factors for post-HSCT PRCA in the multivariate analysis, which was consistent with the multivariate analysis in the nested cohort. We also performed a multivariate analysis in the subgroup of 33 patients with MSD-HSCT (9 patients with PRCA vs. 24 patients without PRCA), no significant difference of ATG for post-HSCT PRCA was observed (**Supplemental Table 3** and **Supplemental Table 4**).

**Table 4:** Univariate and multivariate analyses of risk factors for pure red cell aplasia in the entire cohort.

	Univariate analysis			Multivariate analysis		
Univariate analysis	OR	95% CI	P-value	OR	95% CI	P-value
Donor type (HID vs MSD/URD)	0.178	0.064-0.494	<0.001	0.032	0.004-0.244	<0.001
ATG (Yes vs No)	0.324	0.187-0.559	<0.001	NA	NA	0.182
Patient age (≥35 vs <35)	5.782	1.281-26.088	0.022	NA	NA	0.060
DRI-R (High/very high vs Low/Int)	NA	NA	0.120	–	–	–
Donor age (≥37 vs <37)	NA	NA	0.413	–	–	–
Donor-recipient blood type (A–O vs other)	NA	NA	0.814	–	–	–
Donor gender (Male vs female)	NA	NA	0.382	–	–	–
Patient gender (Male vs female)	NA	NA	0.138	–	–	–

DRI-R, refined disease risk index; HID, haploidentical donor; MSD, matched sibling donor; URD, unrelated donor; NA, not available.

### Anti-Donor Isohemagglutionins

At the time of PRCA diagnosis, the median level of IgG anti-donor isohemagglutinins (ISO) in the PRCA cohort was 1:128 (range, 1:1–1:1024). However, IgM anti-donor ISO in patient with PRCA was at a relatively low level (≤1:32). At the time of PRCA recovery, the IgG anti-donor ISO titer was less than 1:64, and the IgM anti-donor ISO titer was less than 1:8. The median decreases in the anti-donor ISO titer index were 0 (range, 0–4) and 2 (range, 0–4) for IgG and IgM, respectively.

**Figure 3:**
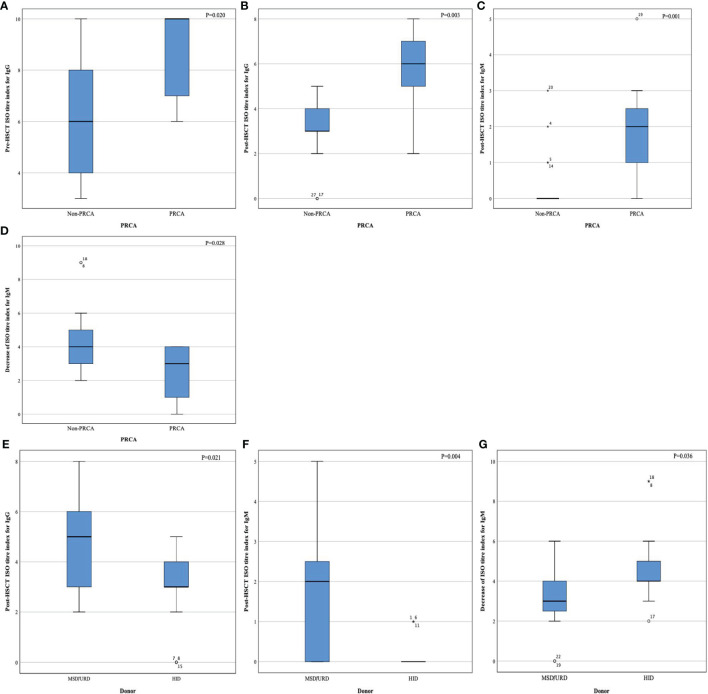
Index of anti-donor isohemagglutinin titer. Pre- HSCT IgG **(A)**, post-HSCT IgG **(B)**, post-HSCT IgM **(C)**, decrease index of IgM after HSCT **(D)**, post-HSCT IgG **(E)**, post-HSCT IgM **(F)**, and decrease index of IgM after HSCT **(G)**.

Patients with PRCA had a higher IgG anti-donor ISO in the first 4 months post-HSCT than patients without PRCA (**Figure 3A**). Both post-HSCT IgM and post-HSCT IgG ISO were more elevated in patients with PRCA than in patients without PRCA (*P*=0.001, **Figures 3B, C**). The disappearance of IgM anti-donor ISO was more significant in patients without PRCA than in patients with PRCA (*P*=0.028, **Figure 3D**). Patients using HIDs had a lower post-HSCT IgM and IgG anti-donor ISO than those using MSDs or URDs (**Figures 3E, F**). IgM anti-donor ISO was observed to significantly decrease in HID-HSCT rather than MSD-HSCT or URD-HSCT (*P*=0.036, **Figure 3G**). No difference in IgG or IgM anti-donor ISO level was found between the groups on the donor blood type (**Supplemental Table 5**).

### Transplantation Outcome

**Figure 4:**
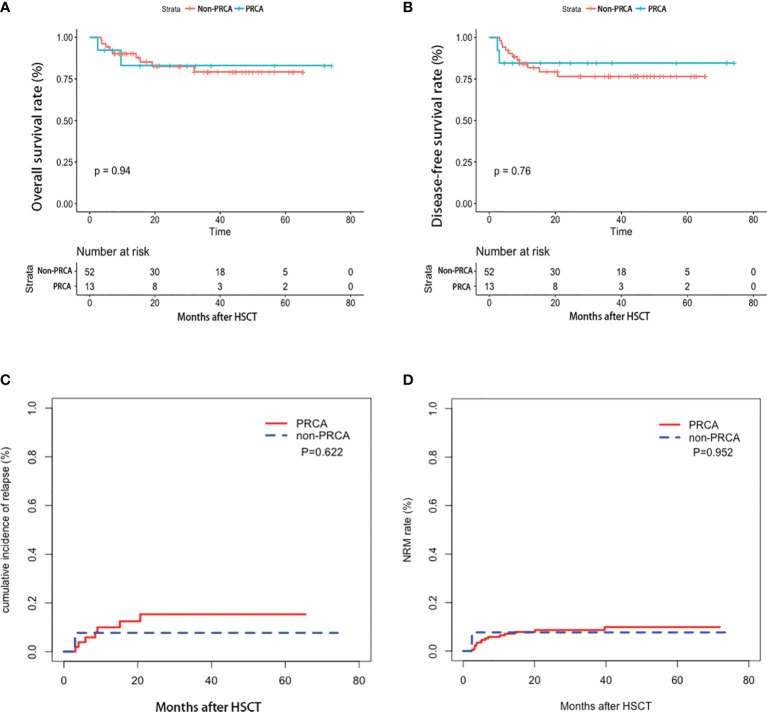
Transplantation outcome in the cohort. Overall survival **(A)**, disease-free survival **(B)**, relapse rate **(C)**, and non-relapse mortality **(D)**.

The median red cell infusions 100 days post-HSCT were 6 (range, 0 to 35) U for patients with PRCA and 4.5 (range, 0 to 33) U for patients without PRCA, respectively. In patients with PRCA, neutrophils and platelets engrafted at a median of 12 (range, 10–53) days and 12 (range, 0–26) days, respectively. In patients without PRCA, neutrophils and platelets engrafted at a median of 12 (range, 7–20) days and 13 (0–33) days, respectively. No difference was observed between the two groups in red cell infusion, neutrophil and platelet engraftment, acute GVHD incidence, and chronic GVHD (**Table 1**). The median survival rates were 24.6 (2.4-74.1) months post-HSCT for patients with PRCA and 27.7 (3.5-65.4) months post-HSCT for patients without PRCA, respectively. **Figure 4** shows the 2-year OS, DFS, relapse rate, and NRM between the two groups, but no significant difference was found. The development of PRCA did not affect the transplantation outcomes in the multivariate analysis using the Cox regression model (**Supplemental Table 6**).

## Discussion

The present study is a prospective nested case-control study aiming to determine the prevalence, donor-related factors, and outcome of PRCA following major ABO-incompatible transplantation. It is revealed for the first time that patients undergoing HID-HSCT may be at a lower risk for developing post-HSCT PRCA than those using MSDs or URDs in major ABO-incompatible transplantation. Meanwhile, the effect of post-HSCT PRCA on transplantation prognosis is not sufficiently significant.

This study revealed that patients with PRCA tend to have a higher level of pre-HSCT anti-donor ISO for IgG and post-HSCT anti-donor ISO for both IgG and IgM than patients without PRCA in the major ABO-incompatible transplantation method. In addition, half of the 13 patients with PRCA had a high IgG ISO level of 1:128 when diagnosed with PRCA initially. This phenomenon may suggest that the development of post-HSCT PRCA may depend on the persistence of anti-donor ISO for the first 4 months post-HSCT, especially for IgG anti-donor ISO. As reported, Bolan et al. revealed that the delayed onset of donor red blood cell (RBC) was associated with the time of anti-donor ISO disappearance, and the time of post-HSCT anti-donor ISO disappearance was significantly linked to pre-HSCT anti-donor ISO titer (6). On the other hand, Griffith et al. found that considerable proportion of residual plasma cells from recipients after transplantation may secrete sufficient antibodies to destroy nascent erythroblast precursors and prevent timely maturation (10). Similarly, a previous study by Gmür showed that donor RBCs engrafted following the decrease in isohemagglutinins titer to below 1:16.^22.^ Another report by Longval et al. also demonstrated that a high level of pre-HSCT anti-donor ISO was associated with increased risk of PRCA (25). Taken together, the primary post-HSCT PRCA could be explained by the high level of IgG ISO at the time of transplantation to some extent. Interestingly, it was found that recipient-derived ISO was expected to disappear within 120 days after HSCT by a poster (26). Correspondingly, our study showed that the median recovery time of primary PRCA was 125.5 days post-HSCT. In addition, several studies found that the rebound in titers of anti-donor ISO during the first 4 to 10 weeks post-HSCT may account for the development of the secondary post-HSCT PRCA (12, 13, 27). This finding coincided with our study that patients with secondary PRCA were identified approximately 60 days post-HSCT. However, our research failed to find a cut-off value of anti-donor ISO to predict the occurrence of PRCA due to the small sample for anti-donor ISO monitoring.

Donor type was the only factor associated with the development of post-HSCT PRCA in our study. Patients using HIDs had a lower cumulative incidence rate of PRCA (0.8%) compared to those using URDs and MSDs (*P*<0.001). Additionally, we observed that patients undergoing haploidentical transplantation appeared to have a lower titer level of anti-donor ISO post-HSCT for both IgG and IgM and a higher disappearance rate of IgM-ISO. Hence, it could be hypothesized that HID-HSCT may be more effective in removing the ISO compared to other transplantation methods. Previous studies have illustrated that the disappearance of anti-donor ISO was more effective in URD-HSCT rather than in MSD-HSCT (49 versus 166 days, *P*<0.001) (13, 20), and the patients who underwent URD-HSCT showed a trend toward a lower incidence rate of post-HSCT PRCA compared to those who underwent MSD-HSCT (9). Consequently, transplantation using major ABO-incompatible grafts from different donor types is possible to have the additional potential of removing anti-donor ISO owing to the discrepancy of graft-versus-host plasma cells effect.

Remarkably, ATG was added to all patients in the haploidentical transplantation in our study, which may confuse the effect of donor type on the development of post-HSCT PRCA. However, ATG was not associated with PRCA in the multivariate analysis. Occasionally, some patients with post-HSCT PRCA could be treated effectively with ATG (28, 29). A recent case series revealed that ATG (equine-ATG, 40 mg/m^2^ for 4 days) was a viable salvage approach for five patients with refractory PRCA associated with ABO-incompatible HSCT (28).

The standard treatment for post-HSCT PRCA remains to be determined. Whether the recovery of patients with PRCA results from self-limited remission or treatment is difficult to distinguish. Hirokawa et al. demonstrated that the intervals of reticulocyte recovery in patients with the additional intervention were similar to those without, which implied the failure to provide supportive evidence on the superiority of treatment (30). Notably, pre-HSCT plasma exchange was confirmed to reduce pre-HSCT ISO titer significantly efficiently, which led to a lower incidence rate of post-HSCT PRCA (3/98 versus 9/55) (9). Therapeutic plasma exchange (TPE) was an effective strategy for post-HSCT PRCA, as reported previously (31–34). Apart from five patients with PRCA receiving transfusion and IVIG only in our study, six patients were treated with TPE additionally and achieved definite treatment effect. In patients with refractory PRCA, daratumumab, eltrombopag, and rituximab may be effective strategies in previous cases (35–38).

This study has some limitations. The two factors we applied to match may not be the best choice in our nested case-control study. Additionally, we only monitored anti-donor ISO titers twice (pre-HSCT and the fourth month post-HSCT) in a small group of 28 patients. It was required that anti-donor ISO be monitored more intensively during the first 4 months post-HSCT and the time when patient received any therapy treatment. Due to the limited group number, it was of difficulty to determine a cut-off value of anti-donor ISO pre-HSCT or post-HSCT to predict the development of post-HSCT PRCA. A larger cohort should be included to observe dynamic changes in isohemagglutinins titers. Moreover, the changes in reticulocyte and donor myeloid chimerism should be monitored regularly, which restricts the ability to reveal the association between donor type and PRCA.

In summary, post-HSCT PRCA is a less threatening complication and is prevalent among a small group after major ABO-incompatible HSCT. Most patients with PRCA could recover within half a year after the diagnosis of the PRCA. In addition, haploidentical donors may help to achieve a relatively low risk of post-HSCT PRCA.

## Data Availability Statement

The original contributions presented in the study are included in the article/**Supplementary Material**. Further inquiries can be directed to the corresponding author.

## Author Contributions

PZ, analyzing data, writing of the original draft. YW, constructing the draft and re-editing of the revised manuscript. JS, YZ, XL, LL recruiting patients, data collecting, and discussing the results. DC and JX, offering the help of testing the anti-donor isohemagglutinins and the data collecting of red cell transfusion. HH, funding acquisition, and project administration. YL, funding acquisition, project administration, review, and validation. All authors contributed to the article and approved the submitted version.

## Funding

This work was supported by grants from the National Natural Science Foundation of China (81970158), the National Key Research and Development Program of China (2018YFA0107804), and the Pediatric Leukemia Diagnostic and Therapeutic Technology Research Center of Zhejiang Province (JBZX-201904).

## Conflict of Interest

The authors declare that the research was conducted in the absence of any commercial or financial relationships that could be construed as a potential conflict of interest.

## Publisher’s Note

All claims expressed in this article are solely those of the authors and do not necessarily represent those of their affiliated organizations, or those of the publisher, the editors and the reviewers. Any product that may be evaluated in this article, or claim that may be made by its manufacturer, is not guaranteed or endorsed by the publisher.

## Supplementary Material

Supplementary Table 1 Characteristics of patients with post-HSCT PRCA.

Supplementary Table 2 Characteristics of the entire cohort.

Supplementary Table 3 Characteristics of patients with MSD-HSCT.

Supplementary Table 4 Univariate and multivariate analysis of risk factors for pure red cell aplasia in 33 patients with MSD-HSCT.

Supplementary Table 5 The level of anti-donor isohemagglutinins.

Supplementary Table 6 Multivariate analysis of risk for transplantation outcomes.

Supplementary Figure 1 Cumulative incidence rates of pure red cell aplasia in the cohort. Donor type **(A)**, Refined Disease Risk Index **(B)**, Donor age **(C)**, and anti-thymocyte globulin **(D)**.
