# Supplementary material for: Prevalence of Pure Red Cell Aplasia Following Major ABO-Incompatible Hematopoietic Stem Cell Transplantation
Source: Front Immunol. 2022 Feb 11;13:829670. doi: 10.3389/fimmu.2022.829670 (PMC8873189; doi:10.3389/fimmu.2022.829670)
Supplement: Supplementary Table 1 — Characteristics of patients with post-HSCT PRCA. [file Table_1.pdf]

Supplemental Table 1. Characteristics of patients with post-HSCT PRCA

	Primary PRCA (N=7)	Secondary PRCA (N=6)	P
Patient age, median (range), years	46 (38–52)	45 (30–55)	0.775
Patient sex, female/male, n	6/1	3/3	0.431
Donor-patient blood type, n			0.764
A–O	4	2	
B–O	3	4	
Disease			0.027
AML/MDS	2	5	
ALL	5	-	
Other	-	1	
Donor type			0.503
HID	1	0	
MSD	5	4	
URD	1	2	
Donor age, median (range), years	40 (14–51)	41 (27-53)	0.721
Donor sex, female/male, n	4/3	2/4	0.764
ATG for GVHD prophylaxis			1.000
Yes	3	3	
No	3	2	
Iso-titers index			
PRCA diagnosis			
IgG	7 (6 –10)	6 (0–9)	0.236
IgM	4 (0–5)	1.5 (0–7)	0.469
PRCA resolution			
IgG	6 (0–6)	3 (0-6)	0.178
IgM	2 (0–3)	0 (0–1)	0.199
PRCA diagnosis time post-HSCT, days	-	60 (45–114)	-
PRCA course, days	125.5 (116–300)	158 (66–777)	0.855

Supplemental table 2. Characteristics of the entire cohort

	Total, n (%)	PRCA, n (%)	Non-PRCA, n (%)	P
Total	187	13	174	
Patient Gender (female/male)	91/96	9/4	82/92	0.155
Patient age, median (range), years	35 (15–67)	46 (27–55)	33 (15–67)	0.007
Disease				0.950
AML	83	7	77	
ALL	69	5	64	
Other	35	1	33	
DRI-R				0.183
Low/ Int	119	11	108	
High / very high	68	2	66	
Donor gender (female/male)	65/122	6/7	59/115	0.371
Donor age, median (range), years	37 (13–56)	40 (14–53)	36.5 (13–56)	0.324
Donor type				<0.001
MSD	33	9	24	
HID	131	1	130	
URD	23	3	20	
Intensity of conditioning regimen				1.000
MAC	179	12	167	
RIC	8	1	7	
ATG for GVHD prophylaxis				<0.001
Yes	163	6	157	
No	24	7	17	
Donor-recipient blood type				0.830
A–O	81	6	75	
Other	106	7	99	
CD34 cell, median (range), $\times 10^6$/kg	5.82 (1.4–20.63)	5.72 (3.05–13.50)	5.81 (1.4–20.63)	0.984
MNC, median (range), $\times 10^8$/kg	14.13 (1.40–55.28)	13.48 (7.56–43.90)	14.17(1.4–55.28)	0.757
Red cell transfusion at 100d, median (range), U	4 (0–56)	6 (0–35)	4 (0–56)	0.264
Acute GVHD at 100d				
Grades II-IV	55	2	53	0.351
Grades III-IV	24	0	24	0.380
Moderate – severe chronic GVHD, n	35	1	34	0.468
Median follow-up (range), Mo	27.1 (2.4–74.1)	24.6 (2.4–74.1)	27.4 (2.4–72.7)	0.875

Supplemental Table 3. Characteristics of patients with MSD-HSCT

Characteristic	PRCA (N=9)	Non-PRCA (N=24)	P-value
Patient sex, female/male, n	7/2	13/11	0.263
Patient age, median (range), years	41 (27–52)	37 (24–67)	0.225
Disease			0.987
AML/MDS	4	11	
ALL	4	10	
Other	1	3	
DRI-R			0.073
Low/Int	9	16	
High/very high	0	8	
Donor sex, female/male, n	4/5	13/11	0.708
Donor age, median (range), years	45 (26–53)	39.5 (25–55)	0.352
Intensity of conditioning regimen			1.000
MAC	8	22	
RIC	1	2	
ATG for GVHD prophylaxis			1.000
Yes	2	7	
No	7	17	
Donor-recipient blood type			0.690
A–O	4	8	
Other	5	16	
CD34+ cells, median (range), $\times 10^6$/kg	6.84 (3.047–13.50)	7.28 (2.44–16.65)	0.544
MNC, median (range), $\times 10^8$/kg	13.66 (10.19–43.90)	18.35 (1.40–30.29)	1.000
Red cell transfusion at d100, median (range), U	6 (2–12)	2 (0–56)	0.136
Median follow-up (range), Mo	32.5 (4.6–74.1)	30.0 (2.4–72.7)	0.419

Supplemental Table 4. Univariate and multivariate analysis of risk factors for pure red cell aplasia in 33 patients with MSD-HSCT

	Univariate analysis			Multivariate analysis		
Univariate analysis	OR	95% CI	P-value	OR	95% CI	P-value
ATG (Yes vs No)	NA	NA	0.703	-	-	0.703
Patient age (≥35 vs <35)			0.237			0.237
DRI-R (High/very high vs Low/Int)	NA	NA	0.062	-	-	0.062
Donor age (≥37 vs <37)	NA	NA	0.596	-	-	0.596
Donor-recipient blood type (A–O vs other)	NA	NA	0.492	-	-	0.492
Donor gender (Male vs female)	NA	NA	0.577	-	-	0.577
Patient gender (Male vs female)	NA	NA	0.222	-	-	0.222

Supplement table 5. The level of anti-donor isohemagglutinins

	PRCA			Donor-recipient blood type			Donor type		
	Yes	No	P	A-O	B-O	P	HID	Non-HID	P
N	7	21		12	16		17	11	
IgG-titer index (Median, range)									
Pre-HSCT	10 (6-10)	6 (3-10)	0.020	7 (3-10)	6 (3-10)	0.186	6 (3-10)	7 (3-10)	0.293
Post-HSCT	6 (2-8)	3 (0-5)	0.003	4 (2-6)	3 (0-8)	0.149	3 (0-5)	5 (2-8)	0.021
Decrease index	2 (1-8)	3 (0-10)	0.957	3 (0-8)	1.5 (0-10)	0.312	3 (0-10)	2 (0-8)	0.567
IgM-titer index (Median, range)									
Pre-HSCT	5 (2-5)	5 (2-9)	0.868	5 (3-9)	4 (2-9)	0.109	5 (2-9)	5 (2-6)	0.675
Post-HSCT	2 (0-5)	0 (0-3)	0.001	0.5 (0-3)	0 (0-5)	0.302	0 (0-1)	2 (0-5)	0.004
Decrease index	3 (0-4)	4 (2-9)	0.028	4 (2-9)	4 (0-9)	0.602	4 (2-9)	3 (0-6)	0.036

Supplemental Table 6. Multivariate analysis of risk for transplantation outcomes

	OS	DFS	RI	NRM
	P-value	*P*-value	*P*-value	*P*-value
Donor age<br>(≥37 vs <37)	0.143	0.099	0.306	0.971
Donor gender<br>(Male vs female)	0.696	0.519	0.223	1.000
Donor type<br>(HID vs MSD/URD)	0.933	0.657	0.743	0.813
ATG<br>(Yes vs No)	0.665	0.917	0.547	0.980
DRI-R<br>(High/very high vs Low/Int)	0.125	0.196	0.227	0.399
PRCA<br>(Yes vs No)	0.521	0.808	0.760	0.360

Abbreviation: PRCA pure red cell aplasia; *AML* acute myelocytic leukemia; *ALL* acute lymphoblastic leukemia; *MDS* myelodysplastic syndrome; *DRI-R* refined disease risk index; *HID* haploidentical donor; *MSD* matched sibling donor; *URD* unrelated donor; *GVHD* graft versus host disease; *MAC* myeloablative conditioning; *RIC* reduced intensity conditioning; *HSCT* hematopoietic stem cell transplantation; *NA,* not available,
