# Supplementary figures and images for: Prevalence of Pure Red Cell Aplasia Following Major ABO-Incompatible Hematopoietic Stem Cell Transplantation
Source: Front Immunol. 2022 Feb 11;13:829670. doi: 10.3389/fimmu.2022.829670 (PMC8873189; doi:10.3389/fimmu.2022.829670)

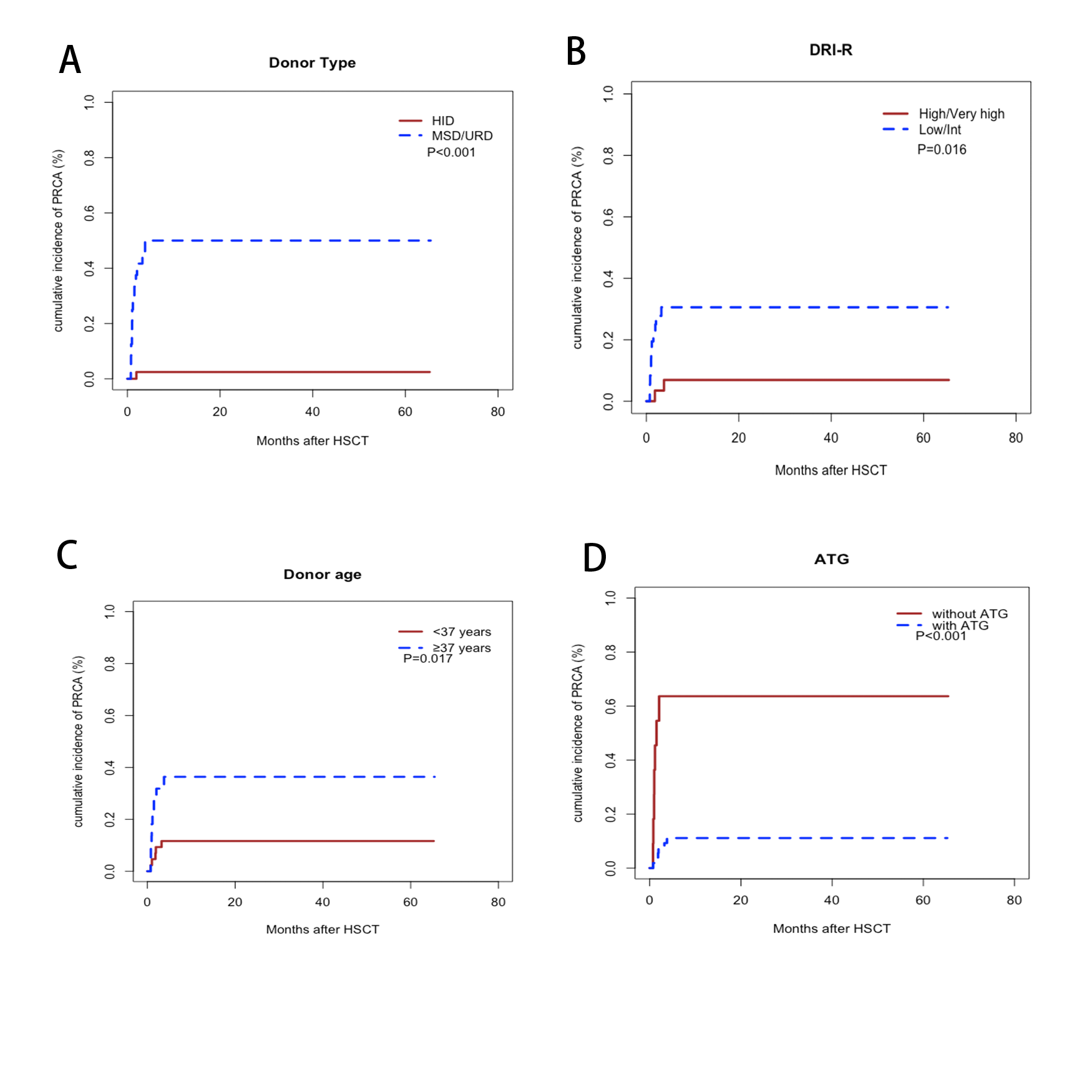

Supplement: Supplementary Figure 1 — Cumulative incidence rates of pure red cell aplasia in the cohort. Donor type (A), Refined Disease Risk Index (B), Donor age (C), and anti-thymocyte globulin (D). [file Image_1.tif]
